# Influence of Gallic Acid and Thai Culinary Essential Oils on Antibacterial Activity of Nisin against *Streptococcus mutans*

**DOI:** 10.1155/2021/5539459

**Published:** 2021-04-24

**Authors:** Pimsumon Jiamboonsri, Pimpikar Kanchanadumkerng

**Affiliations:** ^1^Faculty of Medicine, King Mongkut's Institute of Technology Ladkrabang, Bangkok 10520, Thailand; ^2^Department of Food Chemistry, Faculty of Pharmacy, Mahidol University, Bangkok 10400, Thailand

## Abstract

*Streptococcus mutans* is a well-known oral pathogen commonly associated with a normal dental problem and life-threatening infection. A bacteriocin nisin and the plant-derived compounds including gallic acid (GA) and Thai culinary essential oils (EOs) have been reported to have activity against oral pathogens. However, their synergistic interaction against *S. mutans* has not been explored. The purposes of this study were primarily to investigate anti-*S. mutans* properties and the antibiofilm formation of nisin, GA, and five EOs by using the broth microdilution method. Besides, the morphological change, killing rate, and antibacterial synergism were determined by scanning electron microscopy (SEM), time-kill assay, and checkerboard method, respectively. The results demonstrated that kaffir lime leaf (KLL) oil, lemongrass (LG) oil, and GA showed a potent anti-*S. mutans* activity and inhibited biofilm formation with the possible mechanism targeted on the cell membrane. Additionally, KLL oil revealed anti*-S. mutans* synergism with GA, LG oil, and chlorhexidine with the fractional inhibitory concentration (FIC) indexes ≤ 0.5. Interestingly, GA displayed a high potential to enhance anti-*S. mutans* activity of nisin by lowering the minimum inhibitory concentrations (MICs) to at least 8-fold in a bacteriostatic manner. These results suggest that GA and KLL oil may be potentially used as an adjunctive therapy along with nisin and chlorhexidine to control *S. mutans* infection.

## 1. Introduction

A Gram-positive streptococcal bacterium*, S. mutans*, is an important oral pathogen that can cause common dental caries in humans and life-threatening infectious diseases such as infective endocarditis after entering the blood circulation [1]. To prevent the dental problems and the resulting complication, dental hygiene is necessary throughout the human lifespan [2, 3]. The virulence determinant produced by *S. mutans* is the formation of cariogenic biofilms or dental plaques, which protect the sessile bacteria from antibacterial compounds. Therefore, potential strategies to combat *S. mutans* are the inhibition of biofilm development and eradication planktonic cells [4]. Although mechanical cleaning is an effective approach to remove the cariogenic species and biofilms, the chemical antibacterial agents have been beneficially added to many oral healthcare products for similar purposes [5]. However, long-term use of chemical agents can cause teeth discoloration and disturbance of physiological microbiota [6, 7]. Moreover, the clinically essential antiseptic or antibiotic agents are not worth for treatment of common oral diseases due to an increase in multidrug-resistant bacteria, which is currently one of global health problems [8]. Therefore, natural-derived compounds including polypeptide bacteriocins, phenolic compounds, and herbal EOs became the preference of bacterial controlling agents because of their safety perception and historical usage.

In recent years, a polypeptide bacteriocin, namely, nisin, is attractive as a new generation antibiotic [9]. Nisin produced by safe lactic acid bacteria species *Lactococcus lactis* effectively inhibits food-borne pathogens in both vegetatively growing cells and spores of *Clostridium botulinum* and *Bacillus cereus* [10, 11]. Additionally, this bacteriocin does not change the organoleptic properties of foods [12]. Nisin is therefore approved by the food and drug administration in over 48 countries and becomes widely used as a natural preservative in the various food industries [10]. Despite the fact that nisin is not considered as a common anticaries agent, reports of utilizing nisin to inhibit oral pathogens are still limited [11]. For examples, nisin has demonstrated a potential effect against various cariogenic bacteria including mutans and non-mutans streptococci, *Lactobacillus* sp., and *Actinomyces* sp. [13–15]. Sangcharoen et al.[16] reported that the antibacterial activity of nisin could be enhanced by weak organic acids such as ascorbic acid and citric acid.

GA is a phenolic acid commonly found in several plants [17, 18]. It possesses varieties of health-promoting benefits such as antioxidant, anti-inflammatory, and anticancer, as well as antimicrobial effects [17, 18]. Previous reports showed that GA had strong antimicrobial activities with potentially replacing the synthetic antimicrobial agents for food and biomedical products. Besides, it was documented to have antibacterial potency against *S. mutans* [19, 20]. Therefore, GA becomes an interesting agent used to investigate the enhancement activity of nisin in this study. Apart from using a weak organic acid, the antibacterial efficacy of nisin could be intensified by the addition of plant EOs [21], which are recognized as antimicrobial agents in traditional medicine [22]. The well-known Thai culinary essential oils from finger root (*Boesenbergia pandurate* (Roxb.) Schltr.), kaffir lime (*Citrus hystrix* DC.), holy basil (*Ocimum tenuiflorum* L.), and lemongrass (*Cymbopogon citratus* (DC.) Stapf.) have been reported to contain antimicrobial properties in particular against medically important microorganisms [23–26]. However, research regarding the antibacterial synergism among these EOs and nisin against *S. mutans* is scarce. Therefore, the purpose of this study was primarily to determine the anti-*S. mutans* and antibiofilm activities of nisin, GA, and five Thai culinary EOs. The killing rate and the bacterial cellular morphology were also conducted to investigate the antibacterial characteristics and the possible mechanism, respectively. Moreover, the synergistic interactions among these compounds against *S. mutans* were studies based on a checkerboard microdilution assay to establish the FIC index.

## 2. Materials and Methods

### 2.1. Test Materials

Five EOs including finger root oil (FR, lot. 40017-2019), holy basil oil (HB, lot. 40024-2019), kaffir lime oil (KL, lot. 40020-2019), kaffir lime leaf oil (KLL, lot. 40011-2019), and lemongrass oil (LG, lot. 40003-2019) were kindly provided from Thai-China Flavors and Fragrances Industry Company, Limited. GA (98.8% purity) was purchased from EMD Millipore (Buchs, Switzerland). Chlorhexidine digluconate (20% w/v), nisin from *L. lactis* (1,030,000 IU/g), and saturated alkane standard (C7–C40) were purchased from Sigma-Aldrich (MO, USA). Other chemicals and solvents were of analytical grade and obtained from local distributors.

### 2.2. Analytical Conditions for EOs

The constituents of five EOs were analyzed using a gas chromatography/mass spectrometry (GC/MS-6890n, Agilent, USA) equipped with HP-5 capillary column (30 m, 0.25 mm; J&W Scientific, Folsom, CA). Helium was used as carrier gas at a constant flow rate of 1 mL/min. The oven temperature was initially 50°C for 3 min and then was increased to 200°C at a rate of 10°C/min for 3 min. Finally, the oven temperature was increased to 260°C at a rate of 15°C/min for 20 min. The injector temperature was 250°C. The sample was injected using a split ratio of 1:100. The retention indexes (RI) of constituents were determined with reference to a saturated alkane (C7–C40). Additionally, identification of component was evaluated by computer matching the fragmentation pattern with Wiley 7N spectral library.

### 2.3. Bacterial Strain and Culture Condition

*S. mutans* ATCC 25175 was purchased from the American Type Culture Collection. Three clinical *S. mutans* TLJ1-1, TLJ1-2, and TLJ1-3, which lacked collagen-binding adhesin encoded by the cnm gene [27], were kindly supported by Assoc. Prof. Dr. Jinthana Lapirattanakul, Department of Oral Microbiology, Faculty of Dentistry, Mahidol University, Bangkok, Thailand. This project was approved by the Ethics Committee of Mahidol University (MU-DT/PY-IRB2021/PY022). Bacterial strains were maintained in a mixture of brain heart infusion (BHI; Difco, USA) broth and 20% w/v glycerol at –80°C until use. For experiments, *S. mutans* was grown separately on BHI agar at 37°C for 48 h. The isolated bacterial colonies of actively growing cultures from agar plates were transferred to a test tube with BHI broth and incubated at 37°C for 24 h. The turbidity of inoculum was adjusted spectrophotometrically at 600 nm to obtain an optical density (OD) of 0.2 (approximately 10^6^–10^7^ CFU/mL) before use in the experiments.

### 2.4. Antibacterial Susceptibility Test

The MICs of the test compounds were determined by a broth microdilution method [28]. The test compounds were prepared by dissolving in the BHI medium with Tween 80 (0.04% v/v) and absolute ethanol (0.03% v/v). Then, the serial twofold dilutions of test compounds were mixed with BHI broth at a 1:1 ratio (v/v) in 96-well sterile microtiter plates to obtain final concentrations of 0.006–0.80% v/v for EOs, 0.06–8.00 mg/mL for GA, 31.25–4,000 IU/mL for nisin, and 0.008–1.00 mg/mL for chlorhexidine. 20 *μ*L of the prepared inoculum was added to BHI broth supplemented with the test compounds to obtain a 100 *μ*L final volume in each well. The microtiter plates were then incubated at 37°C for 24 h under aerobic conditions. The negative and positive controls were set in each test. A negative control included the test sample but not the organism, and a positive control included the organism but not the test sample. Chlorhexidine was used as a reference control. The mixture of 0.04% v/v Tween 80 and 0.03% v/v absolute ethanol in BHI medium was also tested to control the effect of solvent. The MIC was defined as the lowest concentration at which no bacterial growth was determined by the unaided eye. The growth endpoint in the wells containing test samples was observed by comparing with the growth in the control wells.

To establish the minimum bactericidal concentration (MBC), 20 *μ*L of each culture medium was removed from wells with no visible growth and placed into 80 *μ*L of sterile BHI broth in 96-well plates. After incubation at 37°C for 24 h, the MBC was determined as the lowest concentration that produced no bacterial growth observed by the unaided eye. Each sample was tested in triplicate in separate experiments.

The enhancing effects of EOs, GA, and chlorhexidine on the antibacterial activity of nisin were also evaluated by the broth microdilution method. The MIC and MBC values of nisin were determined in combination with 0.5×MIC of the test compounds (or 0.4% v/v of EOs when the MIC value of EOs was higher than 0.8% v/v).

### 2.5. Antibiofilm Formation Assay

The effect of test compounds on biofilm formation was determined as described by Wongsariya et al. [29] with modification. The prepared test compounds were mixed in BHI broth supplemented with 1% w/v sucrose by using twofold dilutions method to obtain the final concentrations of 0.006–0.80% v/v for each EO, 0.06–8.00 mg/mL for GA, 31.25–4,000 IU/mL for nisin, and 0.008–1.00 mg/mL for chlorhexidine. 20 *μ*L of the prepared inoculum was added to each well. After incubation at 37 °C for 24 h, the medium was aspirated; the biofilm was then washed twice with 100 *μ*L of sterile saline (0.9% w/v NaCl). The adherent biofilm was fixed with absolute ethanol (100 *μ*L) for 15 min and stained with 0.1% w/v crystal violet for 15 min. After washing the samples with 200 *μ*L of distilled water three times, the dye bound to the biofilm was solubilized by adding 100 *μ*L dimethyl sulfoxide. The extracted dye was measured with a microtiter plate reader (Varioskan LUX, Thermo Fisher Scientific) at the absorbance of 590 nm.

The experiments were carried out in triplicate and the percentages of biofilm inhibition were calculated using

(1) $$\begin{array}{c} \text{biofilm inhibition }\left(\%\right)=\frac{{\text{OD}}_{\left(\text{mean,control}\right)}-{\text{OD}}_{\left(\text{mean,treatment}\right)}}{{\text{OD}}_{\left(\text{mean,control}\right)}}\times 100, \end{array}$$

where OD_(mean,control)_ was defined as the average absorbance of untreated cells, and OD_(mean,treatment)_ was defined as the average absorbance of treated cells. The biofilm inhibition curves were constructed by plotting the percentage of inhibition against concentrations.

### 2.6. Time-Kill Assay

The bactericidal activities of the test compounds were determined according to the time-kill assay of Koo et al. [30] with modification. The bacterial suspension (240 *μ*L, approximately 10^6^ CFU/mL) was added to BHI broth (960 *μ*L) containing the test sample at 1, 2, and 4 × MICs. After incubation at 37°C, sample (20 *μ*L) was collected at different time intervals (0, 2, 4, 8, 12, and 24 h) and a tenfold serial dilution was prepared in sterile saline. Thereafter, 20 *μ*L of each dilution was placed on a BHI agar plate and incubated at 37°C for 24–48 h. A bacterial viability count was performed and recorded as the number of CFU/mL. In each assay, a bacterial growth control was included and consisted of 0.04% v/v Tween 80 and 0.03% v/v absolute ethanol without the addition of test samples. Chlorhexidine was also used as the reference antiseptic agent. All experiments were carried out in triplicate and the experimental results were expressed as mean ± standard deviation (SD). Time-kill curves were established by plotting log_10_ CFU/mL against time. Bactericidal activity was defined as a ≥3 log_10_-fold decrease in the number of survivors at each time point compared with the initial number inoculum.

### 2.7. SEM

The prepared inoculum of *S. mutans* ATCC 25175 was incubated with the test compounds at concentration of 4 × MIC. Bacterial growth controls were performed with the addition of 0.04% v/v Tween 80 and 0.03% v/v absolute ethanol without the test samples and the bacteria treated with chlorhexidine at a concentration of 4 × MIC were used as a reference compound. After incubation at 37°C for 12 h, bacterial cells were collected by centrifugation at 3,000 rpm for 10 min. Then, samples were fixed in 2.5% w/v of glutaraldehyde in 0.1 M phosphate buffer solution (pH 7.2) for overnight and post-fixed in 1% w/v osmium tetroxide in 0.1 M phosphate buffer solution for 1–2 h. The cells were passed through a filter disc (pore size 1.2 micron) and dehydrated using serial concentrations of ethanol (30, 50, 70, 95, and 100% v/v). After critical point drying and coating with a gold sputter, samples were examined using a scanning electron microscope (JSM-IT500HR InTouchScope™, JEOL, Tokyo, Japan).

### 2.8. Checkerboard Microdilution Assay

The antibacterial synergism among test compounds, which could be determined the MIC values, was further studied by checkerboard microdilution assay as previously described by Botelho [31] with modification. 20 *μ*L of the prepared inoculum of *S. mutans* was added to the mixed concentrations of two test compounds, which were in a range of 0.0625–4 × MIC. The experiments were performed in triplicate. The FIC index was defined as the lowest concentration of the combination of test compounds with no visible growth of the test organisms. FIC indexes for the double and triple combinations were calculated using formulas (2) and (3), respectively.

(2) $$\begin{array}{c} \text{FIC index}=\frac{\text{MIC of A in combination}}{\text{MIC of A alone}}+\frac{\text{MIC of B in combination}}{\text{MIC of B alone}}, \end{array}$$

(3) $$\begin{array}{c} \text{FIC index}=\frac{\text{MIC of A in combination}}{\text{MIC of A alone}}+\frac{\text{MIC of B in combination}}{\text{MIC of B alone}}+\frac{\text{MIC of C in combination}}{\text{MIC of C alone}}. \end{array}$$

The FIC index values were interpreted as follows: FIC index ≤0.5; synergistic effect, 0.5 < FIC index <4.0; indifferent and FIC index >4.0; antagonistic effect.

### 2.9. Statistical Analysis

In the time-kill assay, the statistical analyses were performed using SPSS (version 26.0, SPSS Inc., Chicago, IL, USA). An analysis of variance (ANOVA) was performed, and significant differences between means were determined using Tukey's honesty significant difference test or Dunnett's T3 test at a significance level of *p* < 0.05.

## 3. Results and Discussion

### 3.1. Chemical Constituents of EOs

The top ten compositions of five EOs analyzed by GC/MS system are reported in Table 1. Terpenes and terpenoids were the major constituent found in FR (Δ-3-careen, 24.4%), KL (L-limonene, 25.1%), and KLL (citronella, 73.3%) oils, whereas lactone (*γ*-dodecalactone, 33.1%) was mainly found in LG oils. A phenolic compound (3-allyl-6-methoxyphenol, 29.7%) was a major compound in HB oil.

### 3.2. Antibacterial Susceptibility

Table 2 shows the MIC and MBC values of five EOs, GA, and nisin against *S. mutans* ATCC 25175 and three clinical isolates. It was found that LG oil demonstrated the highest potency among EOs with the lowest MIC value of 0.1% v/v, followed by KLL oil (0.8% v/v), whereas FR, HB, and KL oils showed the low potency with MIC and MBC values higher than 0.8% v/v. Therefore, only the effective EOs including KLL and LG were selected to investigate their activities against three clinical isolates. The results indicated the similar efficacy of LG against three clinical strains. KLL showed a higher susceptibility to the clinical strains as shown by the lower MIC and MBC values when compared with those against *S. mutans* ATCC 25175.

For the phenolic compound, GA showed similar MIC and MBC values of 4 mg/mL against both standard and clinical isolates. As shown in Table 2, nisin failed to inhibit the growth of *S. mutans* ATCC 25175 and two clinical isolated at a concentration lower than 4,000 IU/mL, but nisin was able to inhibit one clinical isolate with the MIC and MBC values of 2,000 and 8,000 IU /mL, respectively. This result was inconsistent with the previous report that nisin illustrated the antibacterial activity against *S. mutans* UA 159 strain with MIC value in the range of 625–1250 IU/mL [15]. It could be noted that the different antibacterial efficacy of natural compounds could depend on the type of chemical compounds, the mechanism of action, and the strain of the test microorganism [33]. Therefore, the different bacterial strain could possibly explain this phenomenon.

For the reference agent, chlorhexidine demonstrated similar MIC and MBC values against all tested bacteria with a concentration lower than 0.0156 mg/mL. These results indicated similar or higher efficacy of all test compounds against the standard strain than those against the clinical isolates. Therefore, *S. mutans* ATCC 25175 was only used for further studies. The mixture of 0.04% v/v Tween 80 and 0.03% v/v absolute ethanol in BHI medium produced visible turbidity of bacterial growth similar to the positive control against all test strains. This result implied that Tween 80 and absolute ethanol at the tested concentrations had no inhibition effect on bacterial growth. In addition, this result was consistent with the negative control results of the time-kill assay and SEM study.

### 3.3. Antibiofilm Assay

**Figure 1:**
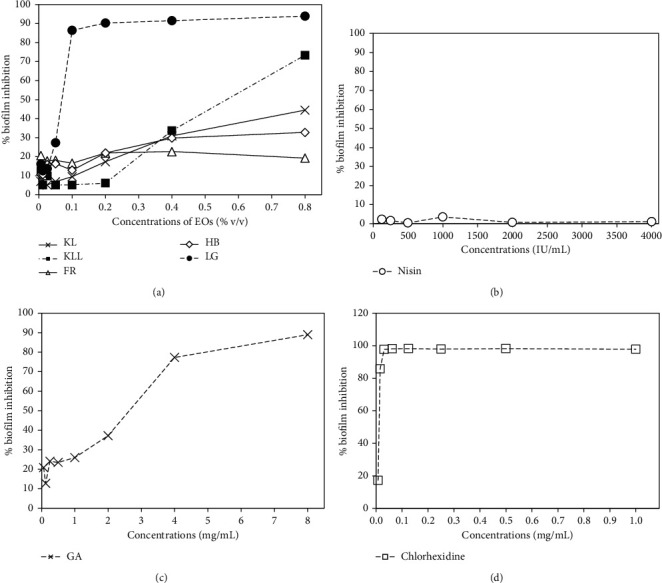
The inhibition effects of five EOs (a), nisin (b), GA (c), and chlorhexidine (d) on biofilm formation of *S. mutans* ATCC 25175.

The antibiofilm formation properties of the test compounds are demonstrated in Figure 1. The results showed that LG oil had the highest antibiofilm activity with greater than 86.44% inhibition when treating at the concentration range of 0.1–0.8% v/v. In the case of KLL oil, the biofilm formation of *S. mutans* was inhibited in a concentration-dependent pattern and showed the maximum inhibition of >73.32% after treatment at 0.8% v/v. This finding was similar to the study of Wongsariya et al. [29] where the antibiofilm formation efficacy of KLL oil was greater than 90% inhibition after treatment at the MIC.

On the other hand, although HB, FR, and KL oils could not determine the MIC in the concentration range of 0.006–0.8% v/v, these oils could inhibit the biofilm formation less than 50% in the same concentration range (Figure 1(a)). This result indicates that the biofilm suppression of EOs may not relate to their antibacterial activity. The composition of EOs was a complex mixture of compounds in different amounts; however, terpene represented the biggest composition along with other non-terpene compounds [34]. Similarly, the major terpene and terpenoid constituents of LG oils were neral dimethyl acetal, citral, and (Z)-citral about 51%, whereas KLL oil mainly consisted of citronella approximately 73% (Table 1). It has been documented that monoterpene-based oils were able to cause a loss of membrane integrity of biofilm cells; therefore the target sites for EOs seemed to be the cell membrane [35]. In addition, bacterial cells with damaged membranes often fail to attach and form biofilm structures [36]. Therefore, KLL and LG oils containing terpenes as a major constituent may be diminished the biofilm formation by these mechanisms [37]. As shown in Figures 1(b) and 1(c), nisin at all test concentrations could not inhibit the formation of biofilm of *S. mutans*, whereas GA at the concentration of 8 mg/mL interfered the formation of biofilms with 88.98% inhibition. Besides, chlorhexidine at a concentration of ≥0.0156 mg/mL could prevent the biofilm formation greater than 85.75% (Figure 1(d)).

### 3.4. The Enhancing Effects of EOs, GA, and Chlorhexidine on the Antibacterial Activity of Nisin

The enhancing activities of test compounds on the antibacterial activity of nisin are shown in Table 3. It was found that the addition of either KLL oil at 0.4% v/v or GA at 2 mg/mL led to a dramatic decrease in the MIC of nisin. The addition of KLL oil was able to reduce the MIC of nisin from >4,000 IU/mL to ⩽31.25 IU/mL (at least 125-fold), whereas the addition of GA showed the capacity to decrease the MIC of nisin to 500 IU/mL (at least 8-fold). Moreover, GA at the concentration of 2 mg/mL demonstrated the capacity to reduce the MBC of nisin by at least 4-fold. It is known that nisin is stable in an acidic medium [38], and GA is a weak acid with pKa values of 4.0 (carboxylic acid). Therefore, this synergistic antimicrobial activity against *S. mutans* could be explained by the decrease of the pH in solution resulting in the increasing solubility of nisin. In addition, it could be similar to ascorbic acid, which enhanced the antibacterial activity of nisin by binding on nisin molecule to stabilize its structure [16, 39]. However, the addition of other EOs and chlorhexidine at sub-MIC values of 0.4% v/v and 0.0078 mg/mL, respectively, demonstrated no enhancing effects on the antibacterial activity of nisin. These results imply that the intensifying ability of EOs and chlorhexidine on the antibacterial activity of nisin against *S. mutans* may not be associated only with their antibacterial activity. Although KLL oil showed a higher ability to reduce the MIC value of nisin greater than GA, only GA displayed the capacity to decrease the MBC value of nisin. Therefore, the combination of nisin and GA was selected to further determine the killing rate and to observe the morphological change.

### 3.5. Time-Kill Assay

**Figure 2:**
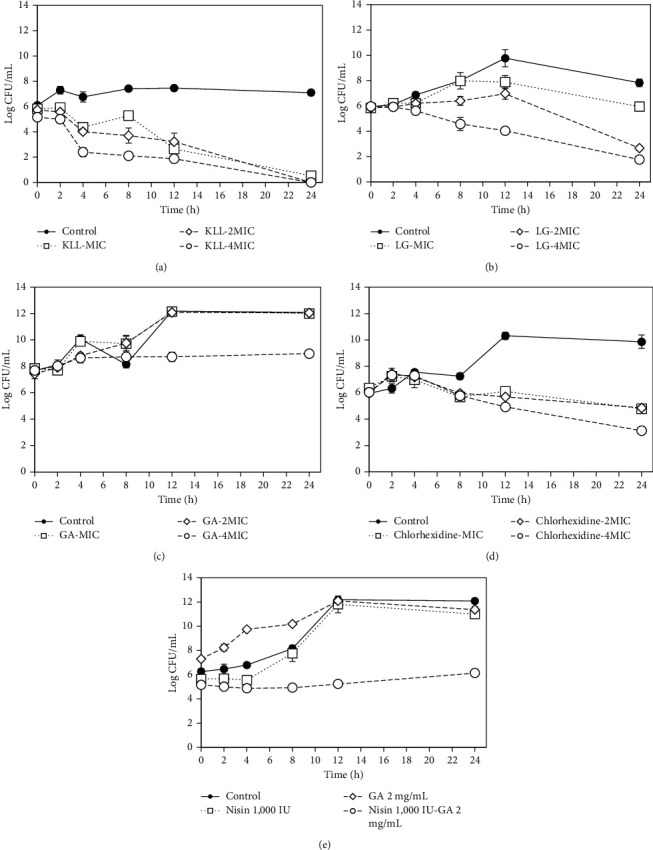
Time-kill curves of *S. mutans* ATCC 25175 after treatment with KLL oil (a), LG oil (b), GA (c), chlorhexidine (d), and the combination of nisin and GA (e). Each symbol indicates the mean ± SD of triplicate samples.

The bacteriostatic and bactericidal effects of the test compounds against *S. mutans* are shown in Figure 2. The ability of KLL oil to kill *S. mutans* was in a time-dependent manner. Treatment with KLL oil at concentrations of 1, 2, and 4×MICs could reduce the number of survival bacteria greater than 3 log_10_ CFU/mL within 12 h and induced complete bacterial cell death within 24 h (Figure 2(a)). The bactericidal activity of LG oil against *S. mutans* was in concentration- and time-dependent pattern. Treatment with LG oil at the concentrations of 2 and 4×MICs exerted the most bactericidal activity (≥3 log_10_-fold decreases) within 24 h, but LG at the concentration of MIC only suppressed the number of survival *S. mutans* constantly for 24 h (Figure 2(b)). Although GA showed the bacteriostatic effect at a concentration of 4×MIC by preventing bacterial growth for 24 h, it failed to inhibit the cell growth at either concentration of 1 or 2×MIC (Figure 2(c)). This result was in agreement with the study of Kang et al. [40] where GA was able to prevent the growth of periodontal bacteria in a bacteriostatic characteristic.

Because the samples at each time point were diluted with normal saline and transferred to a new BHI agar plate, the survival of adaptive isolates may occur from a dynamic manner of the time-kill assay [41, 42]. Therefore, the MIC and MBC values obtained from a static view of broth microdilution assay may not agree with the time-kill assay for a bacteriostatic agent [28]. In the case of chlorhexidine, a bactericidal effect with ≥3 log_10_-fold reductions was observed after treatment at 4×MIC for 24 h, whereas a bacteriostatic effect with <2 log_10_-fold reductions was detected after treatment at either 1 or 2×MIC (Figure 2(d)).

Despite the fact that the ability of plant-derived compounds to potentiate an antibacterial property of bacteriocin has been reported in particular against food-borne pathogens including *Listeria monocytogenes* and *Salmonella* sp. [43], the antibacterial synergism of GA and nisin against oral pathogen was studied using time-kill assay as shown in Figure 2(e). Treatment with the combination of GA and nisin at sub-MIC value was able to suppress the bacterial growth for 24 h, whereas treatment with either GA or nisin alone failed to inhibit bacterial growth. This result indicated that GA could intensify the antibacterial activity of nisin in a bacteriostatic manner.

### 3.6. SEM

**Figure 3:**
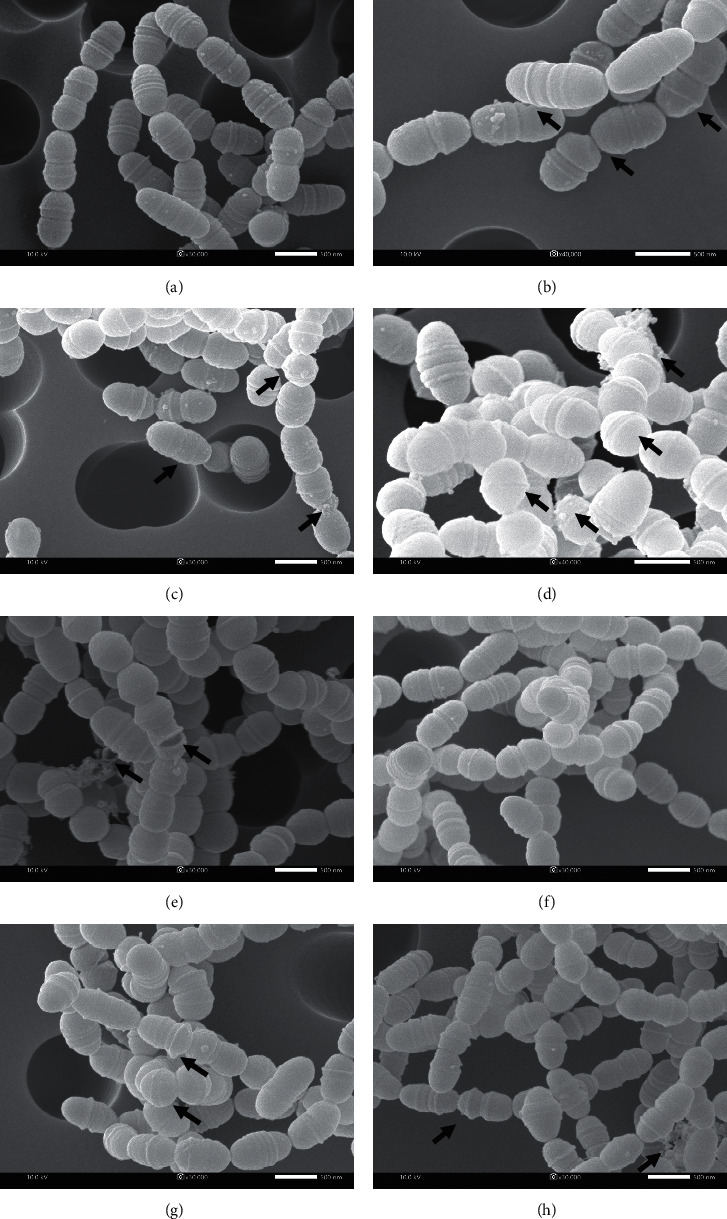
Scanning electron micrographs of *S. mutans* ATCC 25175 at 12 h after treatment with control solvent (0.04% v/v Tween 80 and 0.03% v/v absolute ethanol (a), KLL oil at 4×MIC (b), LG oil at 4×MIC (c), GA at 4×MIC (d), chlorhexidine at 4×MIC (e), nisin at 1,000 IU/mL (f), GA at 0.5×MIC (g), and the combination of nisin at 1,000 IU/mL and GA at 0.5×MIC (h).

SEM images of *S. mutans* ATCC 25175 are shown in Figure 3. The SEM images revealed that KLL and LG oils at a concentration of 4×MIC induced alteration in cell morphology. Control cells in the presence of 0.04% v/v Tween 80 and 0.03% v/v absolute ethanol showed an oval shape with a smooth cell surface (Figure 3(a)). In contrast, cells treated with either KLL oil or LG oil at 4×MIC displayed irregular oval shape with a concavity on the cell surface (Figures 3(b) and 3(c), arrows). In accordance with the previous study of Guimarães et al. [44], *Escherichia coli* treated with citronellol demonstrated irregular cell size with the rough surface. Therefore, the anti-*S. mutans* activities of KLL and LG oils could be principally attributed by terpene constituents along with the contribution of other compounds.

Additionally, the treatment of GA at 4×MIC caused the unseparated spherical cells with coating materials (Figure 3(d), arrows). Apart from lowering environmental pH, GA could also act as a sequestering agent of divalent ions and consequently caused a disruptive effect on the cell membrane [45]. This explanation supports the morphological results of SEM. Similar results could be observed after treating *S. mutans* cells with *Galla chinensis* extracts, which are rich in GA content [46]. The bacterial cells also displayed irregular cell wall structure and showed fewer cells in the chain [46]. In addition, the antibacterial mechanism of GA was suggested to interfere biofilm composition and structure, inhibit glucosyltransferase activity, and directly suppress bacteria growth [18, 47, 48]. When *S. mutans* was treated with chlorhexidine, perforation on the cell surface was observed (Figure 3(e), arrows).

As shown in Figures 3(f) and 3(g), cells treated with nisin (1,000 IU) or GA (0.5×MIC) alone showed similar shape and cell membrane to the control, which was consistent with the results from the time-kill assay. Although nisin was known to play an important role in pore-forming on bacterial membrane [49], the damaged cell membrane could not be observed after treatment with nisin at 1,000 IU. However, after treatment with the combination of nisin and GA, cells showed a small unseparated oval shape with the presence of cellular matrix (Figure 3(h)). The enhanced effect of GA on the antibacterial activity of nisin may be similar to the synergistic effect of citric acid that the combination of nisin and citric acid could control the growth of *S. aureus* and *L. monocytogenes* by inducing the release of cytoplasmic constituents including ions, DNA, and RNA [50].

### 3.7. Checkerboard Microdilution Assay

The interaction among the test compounds was primarily studied using the checkerboard assay. In addition to lowering MIC of nisin, KLL oil together with LG oil, GA, and chlorhexidine demonstrated the synergistic effect against *S. mutans* with the FIC indexes lower than 0.37 as shown in Table 4. However, the additive interaction was found in the triple combination of KLL oil, LG oil, and chlorhexidine. This result was not surprising because LG combined with chlorhexidine showed an indifferent effect with the FIC index of 0.56. Although GA could decrease the MIC of nisin against *S. mutans*, the combination of GA with LG oil or chlorhexidine showed the indifferent effect with the FIC indexes greater than 2. However, no antagonistic effect was observed among these compounds. Because EOs are plant-based products containing a complex mixture of substances, the interaction within each constituent could lead to additive, synergistic, and antagonistic effects [22]. Therefore, the mode of action associated with each constituent should be further investigated.

## 4. Conclusions

GA and two Thai culinary EOs including KLL and LG oils exhibited potent inhibitory effects against *S. mutans* and biofilm formation. The damage to cell membranes resulting in an alteration in bacterial cell morphology was the possible mechanism of action. Despite a weak antibacterial activity of nisin against *S. mutans*, the addition of GA displayed markedly capacity to enhance the anti-*S. mutans* activity of nisin with the bacteriostatic character by 8-fold decrease in MIC values. Additionally, KLL oil combined with LG oil, GA, and chlorhexidine revealed the synergistic antibacterial interaction. Therefore, these results may prove valuable information that GA, and KLL could be potentially utilized as an adjunctive therapy with nisin or chlorhexidine for overcoming *S. mutans*-associated infection.

## Figures and Tables

**Table 1:** Top ten chemical constituents in five Thai culinary EOs.

EOs				Plants				
				*Boesenbergia pandurate* (Roxb.) Schltr.	*Ocimum tenuiflorum* L.	*Citrus hystrix* de Candolle		*Cymbopogon* (DC.) *citratus* Stapf.
Families plant parts				Zingiberaceae	Lamiaceae	Rutaceae		Gramineae
				Rhizome	Leaf	Peel	Leaf	Leaf
No.	Compounds	RI^a^	RI^b^	Chemical composition (% of total)				
1	*α*-Pinene	937	937	1.1		3.2		
2	Camphene	967	979	7.1				
3	Sabinene	976	976				3.0	
4	*β*-Pinene	977	977			19.2	1.1	
5	6-Methyl-5-hepten-2-one	988	988					1.5
6	*β*-Myrcene	992	992					5.1
7	*α*-Terpinene	1007	1007			5.1		
8	Δ-3-Carene	1011	1052	24.4				
9	Limonene	1020	1020			25.1		
10	*γ*-Terpinene	1031	1028			6.1		
11	1,8-Cineole	1036	1036	17.3				
12	*cis*-Ocimene	1038	1040	4.8				
13	Linalool oxide	1074	1034			1.6		
14	*α*-Terpinolene	1079	1063			4.5		
15	Linalool	1099	1101	2.4			3.9	
16	*α*-Terpinolene	1100	1100					0.9
17	-(-) Isopulegol	1152	1152			2.0	2.6	
18	Citronellal	1153	1160				73.3	
19	Camphor	1154	1154	21.8				
20	Neoisopulegol	1156	1164				2.1	
21	Terpinen-4-ol	1165	1163			11.4		
22	Borneol	1173	1173		0.8			
23	*α*-Terpineol	1189	1186	1.0		10.5		
24	Pulegone	1203	1193					1.3
25	*β*-Citronellol	1228	1230				4.3	
26	(Z)-Citral	1248	1249					13.6
27	Geraniol	1255	1258	13.2				3.4
28	*cis*, *trans*-2-Ethylbicyclo [4.4.0] decane	1276	1936				1.4	
29	Citral	1278	1278					15.1
30	Neral dimethyl acetal	1300.4	1334					22.7
31	Citronellyl acetate	1341	1341				5.6	
32	3-Allyl-6-methoxyphenol	1365	1367		29.7			
33	Methyl cinnamate	1379	1395	3.7				
34	Geranyl acetate	1382	1384					1.3
35	*β*-Elemene	1391	1402		10.1			
36	Methyl eugenol	1402	1411		23.2			
37	*trans*-Caryophyllene	1419	1437		25.1			
38	*α*-Humulene	1472	1470		1.4			
39	Germacrene D	1482	1356		1.8			
40	*α*-Selinene	1494	1522		1.3			
41	*β*-Selinene	1500	1511		0.9			
42	Caryophyllene oxide	1581	1673		0.8			
43	*γ*-Dodecalactone^c^		1334					33.1
44	2-Fluoro-4-(4'-propyl [1,1'-bicyclohexyl] -4-yl) Benzonitrile^c^		2701				1.06	

^a^Retention index documented in PubChem database or reported by Babushok et al. [32]. ^b^Retention index relative to a saturated alkane on the HP-5 column. ^c^Tentative identification based on only mass fragmentation.

**Table 2:** Antibacterial activities of EOs, GA, and nisin against *S. mutans* ATCC 25175 and three clinical isolates.

Test compounds	*S. mutans*							
	ATCC 25175		TLJ1-1		TLJ1-2		TLJ1-3	
	MIC	MBC	MIC	MBC	MIC	MBC	MIC	MBC
EOs (% v/v)								
FR	>0.8	>0.8						
HB	>0.8	>0.8						
KL	>0.8	>0.8						
KLL	0.8	>0.8	0.1	0.2	0.8	>0.8	0.4	0.4
LG	0.1	0.1	0.1	0.1	0.1	0.1	0.1	0.1
GA (mg/mL)	4	4	4	4	4	4	4	4
Nisin (IU/mL)	>4,000	>4,000	>4,000	>4,000	>4,000	>4,000	2,000	8,000
Chlorhexidine (mg/mL)	0.0156	0.0156	0.0125	0.0125	0.0125	0.0125	0.005	0.005
Solvent^a^	NE	NE	NE	NE	NE	NE	NE	NE

^a^The solvent was the mixture of 0.04% v/v Tween 80 and 0.03% v/v absolute ethanol in BHI. NE: no antibacterial effect.

**Table 3:** The enhancing effects of five EOs, GA, and chlorhexidine on the anti-*S. mutans* activity of nisin

	Nisin	Combined with						
		FR	HB	KL	KLL	LG	GA	Chlorhexidine
		0.4% v/v	0.4% v/v	0.4% v/v	0.4% v/v	0.05% v/v	2 mg/mL	0.0078 mg/mL
MIC (IU/mL)	>4,000	>4,000	>4,000	>4,000	⩽31.25	>4,000	500	>4,000
MBC (IU/mL)	>4,000	>4,000	>4,000	>4,000	>4,000	>4,000	1,000	>4,000

**Table 4:** FIC indexes of the combination of chlorhexidine, GA, and EOs against *S. mutans* ATCC 25175.

Compounds	MIC		Combined compounds	MIC		FIC index	
	Alone	Combined		Alone	Combined		
KLL (% v/v)	0.8	0.1	LG (% v/v)	0.1	0.025	0.37	SYN^∗^
	0.8	0.1	GA (mg/mL)	4	0.5	0.25	SYN^∗^
	0.8	0.2	Chlorhexidine (mg/mL)	0.0156	0.0019	0.37	SYN^∗^
	0.8	0.05	LG (% v/v)+	0.1	0.025	0.56	IND^∗^
			Chlorhexidine (mg/mL)	0.0159	0.0039		

LG (% v/v)	0.1	0.1	GA (mg/mL)	4	4	2.00	IND^∗^
	0.1	0.025	Chlorhexidine (mg/mL)	0.0156	0.0039	0.56	IND^∗^

GA (mg/mL)	4	8	Chlorhexidine (mg/mL)	0.0156	0.0019	2.12	IND^∗^

^∗^IND: indifferent effect; SYN: synergistic effect.

## Data Availability

The authors declare that all data supporting the findings in this study are provided in the results and discussion section within the article. The datasets used in the current study are available from the corresponding author on reasonable request.
